# Experimental and Numerical Investigation of Mechanical Properties of Lightweight Concretes (LWCs) with Various Aggregates

**DOI:** 10.3390/ma13163474

**Published:** 2020-08-06

**Authors:** Marzena Kurpińska, Tomasz Ferenc

**Affiliations:** Faculty of Civil and Environmental Engineering, Gdansk University of Technology, 80-233 Gdansk, Poland; tomasz.ferenc@pg.edu.pl

**Keywords:** lightweight concrete, insulation concrete, granulated expanded glass aggregate, modulus elasticity, FEM modelling, insulation concrete

## Abstract

High requirements for the properties of construction materials and activities directed at environment protection are reasons to look for new solutions in concrete technology. This research was directed at solutions affecting the reduction of energy consumption and CO_2_ emissions. The use of lightweight concretes (LWCs) allows one to meet both conditions at the same time. The purpose of the research presented in this paper was to investigate the abilities of using lightweight aggregates (LWAs) of the following types: 2 and 4 mm granulated expanded glass aggregate (GEGA) as ingredients with excellent insulating properties and 8 mm granulated fly ash aggregate (GAA) as an ingredient with a relatively high resistance to crushing. The influence of the percentage participation of each aggregate in all LWCs was variable and amounted to 0%, 25%, 50%, 75%, and 100%. A series of 15 LWC mixes were prepared for various LWA participations and for a constant water–cement ratio (w/c = 0.5). Concrete tests were carried out for the following criteria: density, porosity, compressive strength, and the modulus of elasticity. In order to fully analyze fracture processes in LWCs with the participation of GEGA and GAA and to assess the correctness of the results obtained during the experiments, numerical models that corresponded to both geometrical and load diagrams of elements under research were created. The numerical analyses of the LWCs were conducted by means of the conventional finite element method (FEM).

## 1. Introduction

For the past few years, the construction industry had implemented initiatives aimed to contribute to sustainable development. In the production of construction materials, for instance, one way to do so is to increase the usage of artificial lightweight aggregates (LWAs) received from industrial waste. For the production of artificial LWAs, fly ash, cullet, or waste aggregate are used. As an alternative to applying natural raw materials, this type of production contributes to natural deposit protection. Another important aspect in environmental care is that of transportation issues. In some regions with shortages of raw materials, transporting aggregates from distant places generates not only costs but also leads to increasing CO_2_ emissions. Moreover, the production of LWAs makes it possible to reduce landfill waste. Unfortunately, the production of LWAs is still a relatively insignificant element of construction branches in many countries. 

The possibilities of waste material application on a large scale in the industry are being widely investigated nowadays. They have particular significance in civil engineering, where the ability to produce new composites is crucial [1,2,3,4,5]. Lightweight concrete applications in construction reduce building costs, ease construction, and let one take advantage of the use of a relatively ‘green’ building material [6,7,8]. Nowadays, various lightweight concrete types have been invented and proposed for implementation at construction sites. Expanded clay, shale, or glass, as well as natural porous materials like vermiculite or pumice, are typically used as aggregates in lightweight concrete mixtures [9]. Lightweight concrete made from expanded glass granules is one of the latest types of concrete. It is made by incorporating expanded glass granules into the cement paste matrix. The density of expanded glass can vary from 480 to 1600 kg/m^3^ [10]. A specimen of lightweight concrete with an expanded clay aggregate’ density could be 1.6 times higher than a concrete specimen made with expanded glass aggregates [11]. However, Wasserman and Bentur showed that the same aggregate density does not lead to the same concrete strength [12]. Traditional lightweight concrete applications in the construction industry are based on using precast lightweight concrete blocks made from expanded clay aggregates or foamed concrete blocks. Additionally, cast-in-place lightweight concrete applications have become popular in recent years. A lightweight concrete made from expanded glass granules allows for both casting types, and it could be used as both pre-cast concrete for the production of blocks and as cast-in-place lightweight concrete. Cast-in-place concrete provides a much wider range of concrete applications, e.g., wall structures, floor and road thermal insulation, and floor and roof screeds [13,14,15]. Light composites with foamed aggregates have extremely good thermal and acoustic insulation, fire resistance, and resistance to earthquakes compared to traditional concrete [16,17,18,19,20,21,22,23]. The good workability and fluidity of the concrete mix gives the opportunity to form unrestricted shapes. However, the characteristic properties of foamed and porous aggregates require an alternative approach to the mix design and technology of concrete. The mechanism of strength development of a lightweight concrete (LWC) needs to be considered, as does the influence of quantitative and qualitative matrices, and light aggregates to guarantee satisfactory cooperation [24,25,26,27,28,29,30]. 

Regarding the recycling of the construction materials, several investigations have been performed by different researchers. Among these investigations, a model of recycling of lightweight concrete with aggregates containing expanded glass has been proposed. The researchers concluded that recycling of concrete made from lightweight aggregates prolongs the life cycle of the material. Waste concrete can be brought back to life as a product, and it is also possible to innovate production processes by incorporating concrete waste material with expanded glass aggregate [31]. 

Due to the low density of expanded glass granules, the compressive strength of this material is relatively low. For concrete made with aggregates with a density of less than 1000 kg/m^3^, the elastic modulus and compressive strength are strongly affected by the volume fraction of the aggregate. Producers of lightweight expanded glass granules have indicated a compressive strength from 0.45 to 0.55 MPa. By incorporating such a material in a cement matrix, compression strength could be limited due to the expanded glass granules’ compressive strength. Lightweight aggregate outer shell thickness, macro porosity, and broken grain percentage all affect the aggregate strength [32]. The weakest component of a lightweight aggregate concrete is not its cement matrix or interfacial transition zone but the aggregates. Thus, the mechanical performance of lightweight concrete is not only controlled by the cement matrix quality but also the aggregate volume in concrete and the aggregate’s properties [33]. The mechanical properties of the lightweight concrete are therefore ensured by the strength of cement matrix and particle packing. There are two basic ways to increase the compressive strength of this composite material. One of them is to change expanded glass granule volume in the lightweight concrete mixture. Therefore, more strength is obtained by changing the cement paste volume in the concrete mixture design. Another option is improving the lightweight concrete’s mechanical properties by incorporating fine aggregates, such as sand, in the concrete mixture design. The optimal amount of expanded glass aggregate and corresponding strength of cement mortar matrix can be determined, and suitable expanded glass pellet can be chosen for an allotted concrete density and compressive strength [34]. Both solutions affect the thermal properties of the lightweight concrete. In order to increase the mechanical strength, the specimen’s thermal insulation capacity should be slightly reduced because with the increase in density, the compressive strength increases faster than the coefficient of thermal conductivity [35]. Sand and cement paste provide a high thermal conductivity, and, hence, an increase of their volume in lightweight concrete could lead to the improvement of these properties in the final product. To perform research on the thermal conductivity and compression strength changes due to the expanded glass granule volume and sand incorporation effects, both methods were tested. The amount of sand aggregate in lightweight concrete affects its mechanical and physical properties, such as strength, material density and thermal conductivity. The influence of different sand quantities on the properties of lightweight concrete made from expanded glass aggregates was tested. Various lightweight concrete mixtures, including three lightweight concrete mixtures with different expanded glass granule amounts and three lightweight concrete mixtures with different amounts of sand, were prepared to identify the relationships between the mechanical and thermal properties of various amounts of incorporated expanded glass granules and sand in the mixtures. Mechanical and physical properties were tested, and thermal conductivity was observed [36,37,38,39].

The aim of the study was to determine the relationship between the share of lightweight aggregates: granulated expanded glass aggregate (GEGA) and granulated fly ash aggregate (GAA) and their impact on the mechanical properties of the light composite. To achieve this goal, the following tasks were solved: The kinetics of the increase in strength of the tested concrete over time were identified, and the density, strength, and elastic modulus of concrete were determined, depending on the porosity of the composite. In order to fully analyze fracture processes in LWCs with the participation of GEGA and GAA and to assess the correctness of the results obtained during experiments, numerical models that corresponded to both geometrical and load diagrams of the elements under research were created. Numerical analyses of the LWCs were conducted by means of conventional finite element method (FEM) [40,41,42,43,44,45,46]. An approach of modelling the microstructure of LWCs with the distinction of each component, such as mortar and various aggregates, allowed us to assess the stress level in specimens subjected to compressive loading. Furthermore, our numerical simulations could provide significant support in designing LWC components and in determining their mechanical properties, such as the stiffness modulus or strength. 

## 2. Materials and Methods 

### 2.1. Materials

Following [47], CEM I 42.5R Portland cement (Górażdże, Chorula, Poland) was used to perform the tests. The chemical composition and physical properties of the CEM I 42.5R cement are shown in Table 1. 

**Figure 1:**
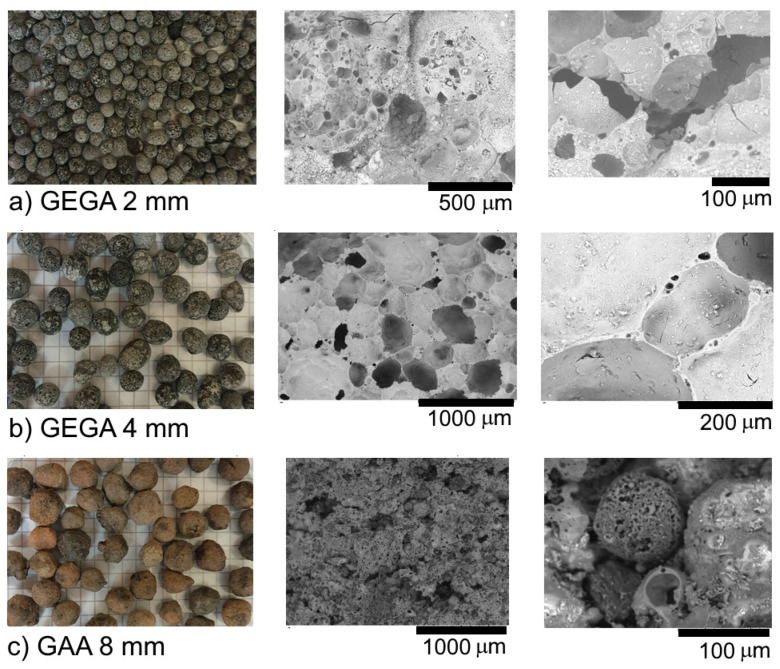
Structure of the aggregates used in the research: (**a**) granulated expanded glass aggregate (GEGA) 2 mm; (**b**) GEGA 4 mm; (**c**) granulated fly ash aggregate (GAA) 8 mm.

GEGAs with 2 and 4 mm grain sizes and the GAAs with an 8 mm grain size (Figure 1a–c) were used as the main components of the LWCs. The chemical composition of the aggregates is given in Table 2. The main component of the aggregates (GEGA and GAA) was SiO_2_ silica, and its content was 33–63% in GEGA and 52.82% in GAA. The physical properties of the aggregates are presented in Table 3.

The porosities of LWAs with the GEGA 2 and 4 and GAA 8 mm grain sizes were examined [50], and the distributions of the GEGA and GAA pore structures are shown in Table 4.

A GEGA and GAA micro-structural analysis was carried out by means of the HITACHI TM3030 electronic scanning microscope (HITACHI, Tokyo Japan), and the results are presented in Figure 1. In case of the GAA grain analysis, one can see that the inner pore diameter was the same as the one of the entire grain cross section. The outer and inner surfaces of the GAA grain were similar in structure. The outer grain surface was an open structure. The GAA grain was characterized by a significantly lower porosity and a higher density than the GEGA grain. The 2 mm GEGA outer grain surface showed a much larger quantity of pores with smaller diameters than that of the 4 mm GEGA. The GEGA outer grain surface was also an open structure that was quite crisp, and cracks were therefore formed on it, which can lead to erosion. The empty spaces and pores of GEGA grain may have been filled with liquids or cement paste. 

### 2.2. Test Methods

The research program included the design and testing of 15 LWC mixtures (1–15). The purpose of the laboratory tests was to determine the influence of grain strength and porosity, as well as the percentage of the LWA content on the properties of the LWCs. An LWC mix of the following composition was designed: 500 kg of CEM I 42.5R and 250 kg of all batch water (free water and moisture in aggregates). The tested aggregate was not pre-moisturized in the laboratory; it contained some amount of natural moisture that had been measured just before mixing. The 2 mm GEGA contained 3.4% natural humidity, the 4 mm GEGA had 2.9% natural humidity, and the 8 mm GAA had 4.1%. The water-to-cement ratio (w/c) 0.5. To maintain a constant w/c, the total water volume was corrected for the moisture contained in the aggregate. No chemical admixtures or additives were used in the test. There were 15 LWC mixtures designed, marked as LWCs 1–15, of different percentage shares of the 2, 4, and 8 mm GEGAs. The percentage shares of each aggregate were designed in the range of 0%, 25%, 50%, 75%, and 100%. The composition of the LWC mixtures is presented in Table 5.

The components of the mixture were mixed in a mechanical mixer. First, cement and water were mixed for 2 min. Then, the aggregate was added to the slurry in accordance with the designed composition of LWCs 1–15 and mixed for another 2 min. Thirty minutes passed after the first contact of the cement with water. Consistency was tested by means of the slump method in accordance with [51]. The concrete mixture was laid in Polyvinyl Chloride (PVC) molds in two layers and vibrated on a vibration table in accordance with [52,53,54]. The compaction of the GEGA concrete mixture had to be carried out so as not to damage the grains of the aggregate. A summary of the specimens for testing is shown in Table 6.

Test specimens were stored for 24 h in molds at the temperature of 20 ± 2 °C, followed by subsequent storage in a chamber with a humidity of 95–100% and a temperature of 20 ± 2 °C, where they were protected against drying. One hour before the test, the specimens were taken out from the chamber and left to dry in the air in the temperature of 20 ± 2 °C, according to [55]. 

**Figure 2:**
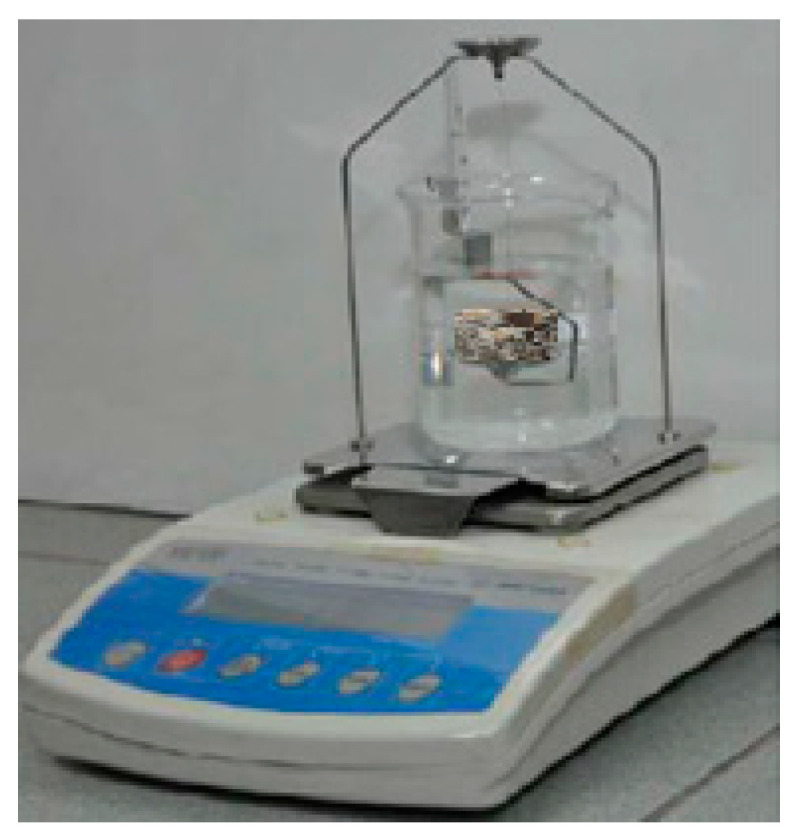
Density measurement by means of the hydrostatic method.

To test density and porosity, a hydrostatic method (also called the Archimedes method) was applied at 28 days. Three specimens of each LWCs 1–15 variant were dried in 105 °C until they reached a steady state. The measuring instruments are shown in Figure 2 (RADWAG AS60/220.R2, Radwag, Radom, Poland). Detailed descriptions of the material properties are shown and discussed in the next chapter.

Density measurement and porosity measurement were performed with the use of 2 × 2 × 2 cm^3^ specimens that were cut out of 10 × 10 × 10 cm^3^ cubes. Each result was an arithmetic mean of three independent mass measurements. The first step was to measure a dry specimen *m_s_*, not saturated with a liquid of any known density. Next, the specimen was saturated with a liquid in a vacuum desiccator to fill all the pores. The mass of the saturated specimen after it had been dried was measured first in the air *m_n_* and then in the liquid *m_w_*. Depending on the composition of the tested LWC, deionized water (*ρ* = 1 (g/cm^3^)) or kerosene (*ρ* = 0.8241 (g/cm^3^)) were taken as the exemplary liquid. Volume density was calculated on the basis of the following equation:

(1) $$d_{p}=\frac{m_{s}}{\left(m_{n}-m_{w}\right)d_{0}},$$

Open porosity (*p_o_*) was calculated on the basis of the equation:

(2) $$p_{o}=\frac{m_{n}-m_{s}}{m_{n}-m_{w}}\cdot 100\%,$$

where *d_p_* is the material’s volume density, (g/cm^3^); *m_w_* is the mass of the specimen when saturated with the liquid, and weighed in a liquid (water), (g); *m_n_* is the mass of the specimen when saturated with the liquid and weighed in the air, (g); *m_s_* is the mass of the dry specimen when weighed in the air, [g]; and *d*_0_ is the liquid density in the tested temperature, (g/cm^3^). Each result is an arithmetic mean of three independent measurements.

The compressive strength of each LWC was tested by means of Advantest 9 Controls machine (Advantest 9, Controls, San Maurizio Canavese, Italy) with a maximum pressure force of 3000 kN. Compressive strength was test according to [56] and was determined as an arithmetic mean of six (tested at 1, 3, 7, 14, 56, and 90 days) or three (at 28 days) measurements, along with the calculation of standard deviation of each batch.

**Figure 3:**
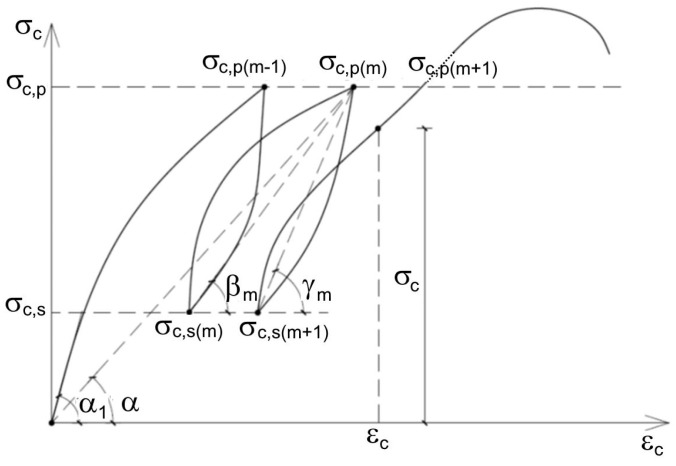
Graph of tension–deformation dependence for lightweight concrete (LWC) under multiple loadings.

The determinations of the modulus of elasticity were conducted according to [57] by using Method A for determining the secant modulus of elasticity in compression. Method A allows one to establish the stress–strain relationship, as well as the initial and stabilized modulus of elasticity, in LWCs. However, cyclic loading is necessary to be applied to specimens. After each cycle of loading, plastic strain decreased and the concrete acquired pseudo-elastic properties. The modulus of elasticity was assessed as a tangent (or secant) to the stress–strain curve in point for the last cycle, when the plastic strain decreased and the LWC acquired linear-elastic properties. (Figure 3.)

**Figure 4:**
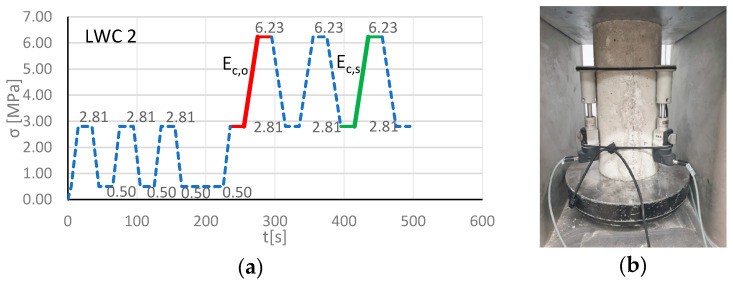
(**a**) The scheme of cyclic loading according to Method A [57] where LWC 2 is shown as an example: $\sigma_{p}=0.5\;\left(\mathrm{MPa}\right),\;\sigma_{b}=2.81\;\left(\mathrm{MPa}\right),\mathrm{and}\;\sigma_{a}=6.23\;\left(\mathrm{MPa}\right)$; (**b**) the specimen during the testing of the modulus of elasticity.

With repeated cyclic loading and unloading, the σ−ε. relationship showed that the plastic deformation $\varepsilon_{p}$ in subsequent cycles decreased. The modulus of elasticity of the concrete could be calculated as the tangent (or secant) of inclination angle to the curve of the m-th cycle. In this paper, the modulus of elasticity for each variant of LWCs 1–15 was determined at 28 days as an arithmetic mean value based on three independent specimen examinations. The secant modulus of elasticity was determined for fifteen specimens (LWCs 1–15) with various amounts of the GEGAs and GAAs. Before the basic examination of the modulus of elasticity, each LWC was tested for its compressive strength *f_cm_* . The secant modulus of elasticity was calculated at the upper stress level, equal to the stress $\sigma_{a}$, up to but no more than 40% of the destructive force. In order to determine those parameters, three cylindrical specimens for each LWC with a diameter of 150 mm and a height of 300 mm (Figure 4b) were examined. The specimens were centrally placed in the testing machine and were unidirectionally compressed with load speeds corresponding to an increase of stress at the level of 0.2 MPa/s. Three extensometers, placed in the center of each specimens, were used to measure strain while measuring the base length of about half of the height of specimens. Method A was used to measure the modulus of elasticity, according to which the specimen was loaded in six cycles; this is presented in Figure 4a, where LWC 2 is shown as an example. During the first three cycles of loading, the specimens were loaded with an $\sigma_{b}=0.1\times f_{cm}$. Then, during the next three cycles, a specimen was loaded between $\sigma_{b}$ and the top stress in the cycle $\sigma_{a}$. The measured values of strain and stress formed the basis for calculation of the initial *E_C_*_,0_ and stabilized *E_C,S_* secant moduli of elasticity according to equations:

(3) $$E_{C,0}=\frac{\sigma_{ma,1}-\sigma_{mb,0}}{\varepsilon_{a,1}-\varepsilon_{b,0}},$$

(4) $$E_{C,S}=\frac{\sigma_{ma,3}-\sigma_{mb,2}}{\varepsilon_{a,3}-\varepsilon_{b,2}},$$

## 3. Results and Discussion

**Figure 5:**
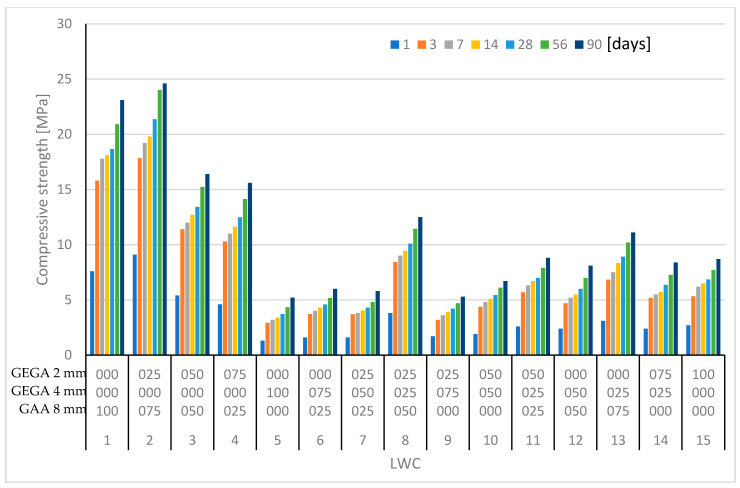
Compressive strength results for LWCs 1–15 at 1, 3, 7, 14, 28, 56, and 90 days.

A summary of mechanical and physical properties of LWCs 1–15 is shown in Table 7, and their compressive strengths at 1, 3, 7, 14, 28, 56, and 90 days are shown in Figure 5. 

**Figure 6:**
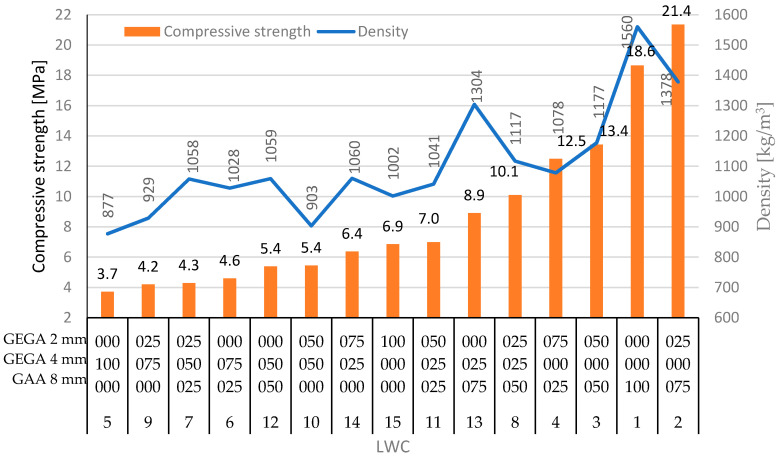
Compressive strength vs. density for LWCs 1–15.

**Figure 7:**
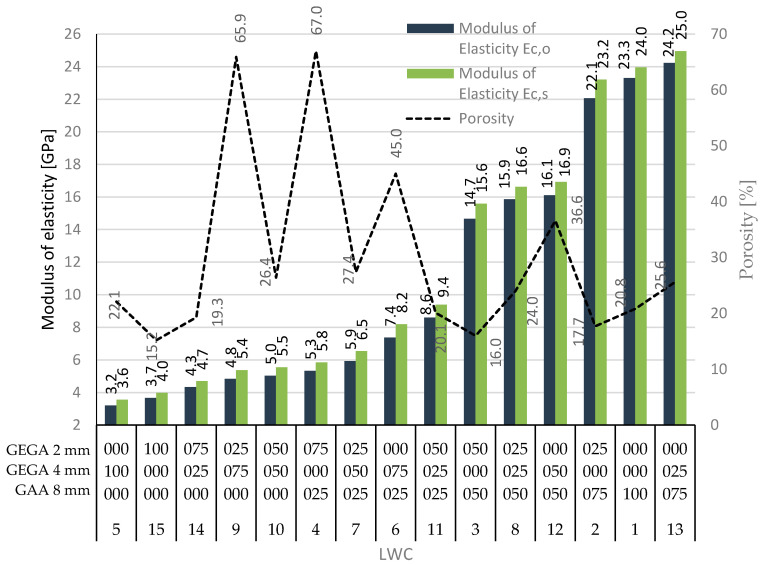
The modulus of elasticity vs. porosity for LWCs 1–15.

The main features of GEGA are its high porosity, low apparent density, and low compressive strength. In general, the reduction of the amount of GEGA and the increasing participation of GAA led to an increase in compressive strength of the LWCs, as well as to an increase of density and the subsequent reduction of the porosity of the composite. Figure 5, Figure 6 and Figure 7 show the test results for LWCs 1–15.

**Figure 8:**
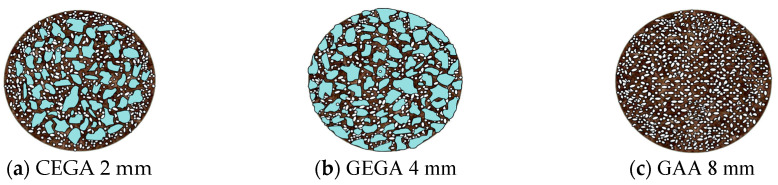
The lightweight grain structure and porosity distribution for: (**a**) 2 mm GEGA, (**b**) 4 mm GEGA, and (**c**) 8 mm GAA.

The most important parameters that determine the properties of light concrete include general porosity and grain density, but the primary ones are lightweight grain structure and porosity distribution. Due to the structure, the three types of lightweight aggregates can be characterized as: 2 mm GEGA (Figure 8a)—aggregates with a diverse pore structure, a predominant number of fine spherical pores, and a smaller number of irregular pores with an open structure smaller than 4 mm; 4 mm GEGA (Figure 8b)—aggregates with a predominant number of irregularly shaped open pores with diameters up to 3.70 mm; and 8 mm GAA (Figure 8c)—aggregates with a predominant number of fine spherical pores with regular shapes and closed structures smaller than 2.83 mm. The structure of LWA grains depends on the production technology and the kind of raw materials used [58].

Though the aggregate density, or porosity, is not directly related to strength, it may be a reliable indicator. This relationship was visible in the case of LWC 5, when 100% 4 mm GEGA were used, and in the case of LWC 9, with 75% of 4 mm GEGA and 25% of 2 mm GEGA. Here, the porosity of the LWC was the highest, and at the same time, the strengths were relatively low—3.6 and 5.4 MPa, respectively. What is more, the highest compressive strength of 21.4 MPa was obtained for LWC 2, which contained 75% 8 mm GAA and 25% 2 mm GEGA, for which the porosity was low and reached 17.7%. On the other hand, the lightweight concrete LWC 15 with the lowest porosity of 15.2% had a compressive strength of 6 MPa, which was only 28% of the highest value of all investigated LWCs. In general, there was some relationship between the compressive strength and density of the LWCs (Figure 6). Moreover, there was also some relationship between the strength and density of the lightweight aggregates and the strength of the LWCs. Due to differences in the structure of the GEGAs and GAAs, a variation in grain strength could be observed. It resulted from the use of various raw materials and different production technologies of the aggregates. In this case, the impact of grain size on strength could be observed as a consequence of both the scale effect and differences in structure. As presented in the conducted research, grains with smaller sizes were characterized by a larger proportion of sintered coating in their volume. As a result, these grains may have exhibited greater stiffness and strength, especially with larger sintered coating thicknesses. The 2 and 4 mm GEGAs could be compared in this instance. In the first case, the crushing strength was 1.4 MPa, while for the second aggregate, it was less than 1.1 MPa. Those strength properties of the aggregates affected the elastic modulus of the LWCs.

Furthermore, formulas for estimating the value of the elastic modulus of a lightweight aggregate can be found [57], and these are based on its grain density or bulk density. By taking the significant differences in the structure of lightweight aggregates with comparable density into account, such a determination of the value of the elastic modulus should be considered as one with a high error potential. Nevertheless, a correlation between density or porosity of the LWC and the modulus of elasticity can be observed (Figure 7), e.g., LWC 1 with a low porosity of 20.8% was characterized by a high modulus of elasticity of 24 GPa, whereas LWC 4 had the highest porosity of 67% and was also characterized by one of the lowest elastic modulus values of 5.8 GPa. Generally, depending on the proportion of the use of GEGA or GAA lightweight aggregates, the LWC modulus of elasticity was determined in the range from approximately 4 to 25 GPa. Therefore, the value of the modulus of elasticity could be several times lower than in the case of normal weight concrete (NWC.) However, when considering the fact that typical cement slurries used both in LWC and in NWC have moduli of elasticity in the range of 12–26 GPa [59], a greater compatibility of slurry deformation with lightweight aggregates could be observed, as compared with natural aggregates. The modulus of elasticity of concrete, which is a two-phase composite material, is dependent on the moduli of elasticity of both components (the lightweight aggregate and matrix), their volume shares, and their adhesion. Therefore, it seems obvious that the modulus of elasticity of concrete with porous aggregates of low modulus is lower than that of NWC. Depending on composition of an LWC, its modulus of elasticity can be about 50–75% of typical values for NWC of the same strength. Other papers have given even lower values; on average, the level of this modulus is about 25–50% for concrete with gravel [58]. In the case of a lightweight aggregate concrete, its modulus of elasticity is determined to a large degree by the elastic properties of the applied aggregate. On the other hand, the influence of the volume proportion between both kinds of aggregates on the modulus of elasticity of the lightweight aggregate concrete is significant. In the presented research, two types of lightweight aggregates (GEGA and GAA) were used. Increasing the GAA volume in the LWA always resulted in the increase of its modulus of elasticity, and on the other hand, an increase of the 4 mm GEGA’s volume in the LWA always resulted in the reduction of its modulus of elasticity.

## 4. FE Modelling and Comparison with Experiment Results

Though fifteen specimens of LWC were examined during the experiments, in the first trial, two specimens were modelled and the results were compared—LWC 1 with 100% GAA and LWC 13 with 75% GAA and 25% 4 mm GEGA—due to the possibility of comparing the effect of the addition of GEGA instead of GAA. 

**Figure 9:**
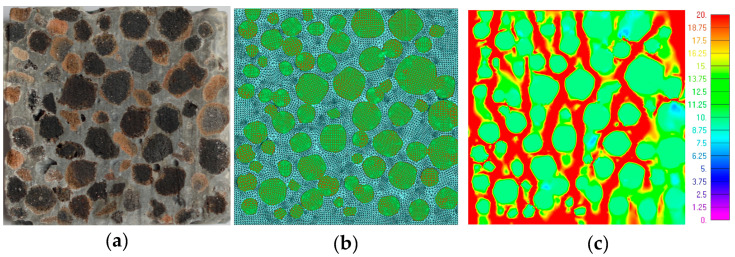
Specimen LWC 1: (**a**) section; (**b**) finite element method (FEM) model; and (**c**) FEM results [MPa].

**Figure 10:**
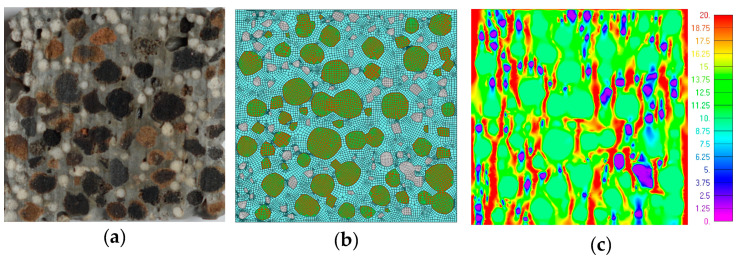
Specimen LWC 13: (**a**) section; (**b**) FEM model; and (**c**) FEM results (MPa).

The geometry of specimen and aggregate decomposition in 2D were assumed after cutting through the middle of the cubic specimen (Figure 9a and Figure 10a). The 2D models were created in FEMAP with the NX Nastran 10.1.1. environment using quadratic and triangle elements of a size about 0.25 mm. The total number of nodes was 15,640 for LWC 1 and 16,081 for LWC 13, while the total numbers of elements were 29,615 and 16,968, respectively (Figure 9b and Figure 10b). The modelled specimens were loaded analogously to the real ones as a unidirectional compression.

Assuming isotropy and elastic–plastic behavior, the material parameters are listed in Table 8.

The results of the FEM calculations are presented in Figure 9c and Figure 10c for the LWC 1 and LWC 13 specimens, respectively. An equivalent stress was found in the moments of the specimens’ destruction. Due to the fact that the level of strength in each component was different, the bearing capacity each specimen was determined by the weakest element. As can be observed, the strength of the GEGA was the least, so the strength of the whole LWC 13 was about twice lower than that of LWC 1 (see Table 7). Analogous results were obtained in FEM modelling. The bearing capacity of the LWC 13 specimen was analyzed, and stress in the GEGA was about 2 MPa (pink and dark blue color). For LWC 1, where the LWC only consisted of GAA, the capacity of the specimen was higher due to the fact that the strength of GAA was 9.8 Pa (green color). Additionally, the modulus of elasticity obtained during numerical simulations was compared with the one from the experiments to check the behavior of the specimens. The modulus of elasticity values (as “E”) were obtained at level E = 21.07 GPa for LWC 1 and E = 19.26 GPa for LWC 13. The values obtained from experiments were 23.3 and 24.2 GPa, respectively. Thus, the relative errors were 9.4% and 20.4%, respectively. 

## 5. Conclusions

The general purpose of the paper was to study the properties of different types of lightweight concretes in order to check the possibility of their application in structural elements as construction materials or as extender and insulation materials. Thus, the study of the mechanical and physical properties of LWC with GAA and GEGA as a natural aggregate substitutes was presented in the paper. Fifteen variations of LWCs with various amounts of aggregates were analyzed and investigated. Physical properties, like porosity and density, and mechanical properties, like compressive strength and the modulus of elasticity, were examined, and the results were compared. Depending on the proportion of the use of lightweight aggregates (2, 4, or 8 mm GEGA), the porosity of LWC was determined in the range from 15.2% for LWC 15 to 67% for LWC 5, the density was in the range from 877 kg/m^3^ for LWC 4 to 1560 kg/m^3^ for LWC 1, the compressive strength was from 3.7 MPa for LWC 4 to 21.4 MPa for LWC 2, and the elastic modulus was from 3.2 GPa for LWC 5 to 24.2 GPa for LWC 13.

Some correlations of the obtained results could be observed. Generally, the impact of the porosity of LWC was gently correlated with its strength—the higher the porosity was, the lower the strength was. This direct relationship could be observed, e.g., for LWC 2 or LWC 1, for which strength was the highest and porosity was relatively low. On the other hand, for LWC 5 and LWC 9, for which the porosity was the highest, the strength was relatively low. Moreover, a correlation between the density of the LWC and the modulus of elasticity could be seen—with the increased density, the modulus of elasticity also increased. That relationship can be seen, e.g., for LWC 1, which was characterized by high density of 1560 kg/m^3^ and the second highest elastic modulus of 23.3 GPa or, on the other hand, for LWC 4 with the lowest density of 877 kg/m^3^ and one of the smallest elastic moduli of 5.8 GPa. However, the relationships with different physical and mechanical parameters determined in the paper were not strict, and only some loose relations could be observed.

Furthermore, the microstructures of the LWCs—aggregates and mortar—were modelled for two chosen LWC types that allowed us to assess the specimens’ behavior under the load. The results of the comparison obtained from computer simulations and the experimental tests showed both qualitative and quantitative convergence. The stress level obtained from computations confirmed the experimental results, namely that the weakest element in the LWC specimens was the lightweight aggregate. The specimens’ destruction occurred due to the stress level being reached in the lightweight aggregate. Hence, the bearing capacity of the whole LWC was determined by the weakest component, which in the LWC was the lightweight aggregate. Moreover, the elastic moduli obtained in experiments and numerical simulations were compared, and the relative error amounted to 20%.

In summary, the lightweight aggregate was the weakest component in concrete, and, hence, it caused a decrease in its mechanical properties like strength or elastic modulus, as compared to normal weight concrete. However, when analyzing other physical properties, like low density or high porosity, the application of LWA also revealed advantages and led to the improvement of the concrete’s properties, e.g., thermal or acoustic insulation. What is more, low density or high porosity led to the self-weight reduction of the structures made from LWC by up to 35%. This allows one to look at the growing use of lightweight concrete next to ordinary one with hope. 

## Figures and Tables

**Table 1:** Chemical composition and physical properties of the CEM I 42.5R cement.

Setting Start Time (min)	Setting End Time (min)		Compressive Strength (MPa)			Blaine Fineness (cm^2^/g)		Loss on Ignition (%)			Water Demand (%)	
			2d		28d							
155	195		30.2		57.3	3504		3.4			27.5	
Content (%)												
**SiO_2_**	**Al_2_O_3_**		**Fe_2_O_3_**	**CaO**	**MgO**	**SO_3_**	**Na_2_O**		**K_2_O**	**TiO_2_**		**Cl**
21.7	6.2		3.1	63.4	1.0	3.9	0.16		0.64	0.25		0.06
Mineralogical composition. content (%)												
**Na_2_Oeq**		**C_3_S**			**C_2_S**	**C_3_A**			**C_4_AF**			
0.7		63.1			7.6	6.1			8.9			

**Table 2:** Chemical composition of the aggregates.

Aggregate Type	Content (%)								
	SiO_2_	Al_2_O_3_	Fe_2_O_3_	CaO	MgO	SO_3_	Na_2_O	K_2_O	Loss on Ignition
GEGA	63.33	0.74	-	14.19	2.98	0.32	13.35	0.57	4.53
GAA	52.82	24.28	7.5	4.5	3.19	0.43	-	0.2	7.1

**Table 3:** Physical properties of the aggregates.

Property		GEGA 2 mm [48]	GEGA 4 mm [48]	GAA 8 mm [49]
Water absorption WA_24_	(%)	15.2	17.8	16.5
Volume density *ρ*_a_	(kg/m^3^)	380	350	1350
Density of dried grain *ρ*_rd_	(kg/m^3^)	340	310	1250
Density of saturated grain *ρ*_ssd_	(kg/m^3^)	360	330	1290
Open porosity P_o_	(%)	37	42	37
Crumble indicator *X_r_*	(%)	22.3	25.9	17.8
pH after 24 h	(-)	11.9	11.9	11.1
Bulk density in a loose state *ρ_b_*	(kg/m^3^)	200	180	680
Thermal conductivity of 40 cm layer of aggregate	(W/m·K)	0.71	0.69	0.85

**Table 4:** Lightweight aggregate granule pore structural analysis.

Granule	Pore Radius (nm)	Pore Volume (cm^3^/g)	Pore Surface Area (m^2^/g)
GEGA 2 mm	1.55–3.71	1.02–7.56 × 10^–^^3^	2.62–12.12
GEGA 4 mm	1.69–3.70	1.25–8.54 × 10^–^^3^	2.99–14.53
GAA 8 mm	1.32–2.83	0.99–6.67 × 10^–^^3^	2.51–10.55

**Table 5:** Details of lightweight concrete mix proportions.

Lightweight Concrete Designation																
LWC		1	2	3	4	5	6	7	8	9	10	11	12	13	14	15
GEGA 2 mm	(%)	0	25	50	75	0	0	25	25	25	50	50	0	0	75	100
	(kg/m^3^)	0	65	130	195	0	0	65	65	65	130	130	0	0	195	260
GEGA 4 mm	(%)	0	0	0	0	100	75	50	25	75	50	25	50	25	25	0
	(kg/m^3^)	0	0	0	0	240	180	120	60	180	120	60	120	60	60	0
GAA 8 mm	(%)	100	75	50	25	0	25	25	50	0	0	25	50	75	0	0
	(kg/m^3^)	580	435	290	145	0	145	145	290	0	0	145	290	435	0	0
CEM I 42,5R	(kg/m^3^)	500														
Water	(kg/m^3^)	250														
(W/C)		0.5														

**Table 6:** The summary of the size and quantity of the specimens for testing.

Test	Specimen Size (m)	Quantity of the Specimens (Pieces)	Total Specimens Quantity (Pieces)
Volume density in a dry state	0.10 × 0.10 × 0.10	3	45
Compression strength at 28 days	0.15 × 0.15 × 0.15	6	90
Compression strength increase at other number of days, than 28 (1, 3, 7, 14, 56, and 90)	0.15 × 0.15 × 0.15	3 × 6 = 18	270
Porosity	0.10 × 0.10 × 0.10	3	45
Modulus of elasticity	ϕ = 0.15, h = 0.3	3	45

**Table 7:** Laboratory tests results for LWCs 1–15.

	Specimens Designation														
LWC	1	2	3	4	5	6	7	8	9	10	11	12	13	14	15
Consistency (mm)	120	110	100	140	90	150	130	130	150	130	120	150	140	70	50
Open porosity *p_o_* (%)	20.8	17.7	16.0	22.1	67.0	45.0	27.4	24.0	65.9	26.4	20.1	36.6	25.6	19.3	15.2
Density *ρ* (kg/m^3^)	1560	1378	1177	877	1078	1028	1058	1117	929	903	1041	1059	1304	1060	1002
Strength f ^cub^_cm 28,_ (MPa)	18.6	21.4	13.4	3.7	12.5	4.6	4.3	10.1	4.2	5.4	7.0	5.4	8.9	6.4	6.9
f ^cyl^_cm 28,_ (MPa)	16.3	18.7	11.8	3.3	11.5	4.3	4.1	10.0	4.1	5.3	6.5	4.9	7.8	6.3	6.0
Modulus of elasticity E_c,0_ (GPa)	23.3	22.1	14.7	5.3	3.2	7.4	5.9	15.9	4.8	5.0	8.6	16.1	24.2	4.3	3.7
E_c,s A_ (GPa)	24.0	23.2	15.6	5.8	3.6	8.2	6.5	16.6	5.4	5.5	9.4	16.9	25.0	4.7	4.0

**Table 8:** Material parameters. (GAA is granulated fly ash aggregate and GEGA is granulated expanded glass aggregate).

Material Parameters		Material			
		GAA 8 mm	GEGA 4 mm	GEGA 2 mm	Mortar
Stiffness modulus	(GPa)	18.9	4.7	3.8	23.9
Poisson ratio	(-)	0.1	0.1	0.1	0.167
Strength	(MPa)	9.8	1.8	1.8	52.8
